# Does quality of care in hip fracture vary by day of admission?

**DOI:** 10.1007/s11657-020-00725-4

**Published:** 2020-03-20

**Authors:** Luke Farrow, Andrew Hall, Lorna Aucott, Graeme Holt, Phyo K. Myint

**Affiliations:** 1 Institute of Applied Health Sciences, School of Medicine, Medical Sciences & Nutrition, University of Aberdeen, Foresterhill, Aberdeen, AB25 2ZD UK; 2 Royal Infirmary of Edinburgh, Edinburgh, UK; 3 Health Services Research Unit, University of Aberdeen, Aberdeen, UK; 4 University Hospital Crosshouse, Kilmarnock, UK

**Keywords:** Hip fracture, Weekend effect, Quality of care, Day of admission, Scottish Hip Fracture Audit, Service provision

## Abstract

**Summary:**

This study investigates if the day of the week a person is admitted with a hip fracture influences the quality of care they receive. We found those admitted Thursday and Friday were likely to obtain poorer postoperative care, indicating a need to optimize services ensuring equality for all.

**Purpose:**

We sought to investigate how the day of admission affects the quality of care provided to hip fracture patients according to national standards (The Scottish Standards of Care for Hip Fracture Patients [SSCHFP]).

**Methods:**

Retrospective analysis of national cohort data. Data were collected by the Scottish Hip Fracture Audit (SHFA) local audit co-ordinators (LACs) at participating Scottish hospitals on behalf of NHS Scotland and the Scottish Government. Adherence to the SSCHFP included assessment of both individual and cumulative standard attainment as a marker for quality of patient care.

**Results:**

From January 2014 to April 2018, 15,351 admissions for hip fracture were recorded. Compared with Monday admission (reference day), patients admitted on a Thursday or Friday had a significantly lower likelihood of achieving the postoperative standards of prompt mobilization (OR 1.77; *p* < 0.001 & OR 1.48; *p* < 0.001, respectively); prompt physiotherapy assessment (OR 8.61; *p* < 0.001 & OR 3.47; *p* < 0.001, respectively); and prompt comprehensive geriatric assessment (OR 1.88; *p* < 0.001 & OR 1.41; *p* < 0.001, respectively). Patients admitted on a Friday or Saturday were less likely to receive the preoperative standards of no delay prior to theatre (OR 1.24; *p* = 0.001 & OR 1.23; *p* = 0.002, respectively) and avoidance of repeat fasting (OR 1.22; *p* = 0.009 & OR 1.22; *p* = 0.01, respectively).

**Conclusion:**

Patients admitted on Thursday or Friday were significantly more likely to not receive postoperative care standards than patients admitted on the reference day (Monday). This appears to be related to inequalities in service provision for Saturday and Sunday compared with the rest of the week.

## Introduction

Managing the complex demands of an increasing hip fracture population is one of the largest challenges facing healthcare providers in the twenty-first century. In the UK, hip fracture incidence is set to rise by up to 75% from 2004 to 2031, with the age and frailty of these patients also likely to rise significantly [1]. Similar trends have been reported for the global hip fracture population [2–4]. A recent global focus on the safety and efficacy of care in this setting has led to the development of national registries and guidelines aimed at standardizing and improving the quality of care for these patients [5, 6].

One such model of care is the Scottish Standards of Care for Hip Fracture Patients (SSCHFP), which was the first set of nationally approved guidelines for hip fracture management [7]. We have recently demonstrated that adherence to these standards is associated with improved outcomes, including reduced mortality, shorter length of hospital stay, and a greater likelihood of discharge back to patients’ premorbid care setting [8].

There has recently been debate and growing interest about the influence of weekend service availability on healthcare outcomes and how the variety of service provision across the week influences patient care [9]. Previous studies have examined how this so called ‘weekend effect’ [9–12], and also day of the week of admission [13], influences mortality and length of stay in hip fracture. Other work has highlighted how the time and day of admission influences delay to surgery [14].

The impact of the day of admission on the overall quality of care that patients receive has, however, not yet been properly assessed or verified. Our primary aim was to assess adherence to the SSCHFP, as a marker for quality of care, in order to examine how the day of admission to hospital influences the quality of care provided to patients with hip fracture. We hypothesized that patients admitted at a weekend (Saturday or Sunday) would have worse quality of care than those patients admitted during the week (Monday to Friday).

## Methods

### Study design, setting, and participants

The study population was drawn from a retrospectively accessed but prospectively collected, validated, and anonymized national audit database – the Scottish Hip Fracture Audit (SHFA). This is collated as part of the Scottish MSk (Musculoskeletal) and Orthopaedic Quality Drive [15] on behalf of NHS Scotland. Data collection includes information from all 22 Scottish hospitals involved in the acute management of hip fracture patients for the purposes of quality improvement, research, and standardization of care. Such data have an established record of use in the examination of pertinent research questions [16, 17] and in annual trend reporting by the Scottish Government [18].

A priori research questions were developed without knowledge of the data, and the authors had no role in patient recruitment. Data collection was performed by Local Audit Co-ordinators (LACs) employed by the individual hospitals.

We included all hip fracture patients aged over 50 years admitted to any of the 22 hospitals which manage acute hip fracture in Scotland between January 2014 and April 2018 that were captured by the SHFA during this period. Outcome data were collected for patients up to 60 days post-admission.

### Scottish Standards of Care for Hip Fracture Patients

The Scottish Standards of Care for Hip Fracture Patients (SSCHFP) is a collection of evidence-based care quality markers against which all patients who are admitted to Scottish hospitals with a hip fracture are measured. These standards were developed by the SHFA advisory group in 2012/13 and first published in 2014. Updated versions contain largely the same standards as first developed baring some minor changes. The 2018 SSCHFP consists of 11 standards shown in Table 1 [7], which have been demonstrated to be associated with improved patient outcomes [8]:

**Table 1:** 2018 Scottish Standards of Care for Hip Fracture Patients

1. Patients with hip fracture should be transferred from the Emergency Department (ED) to the orthopaedic ward within 4 h 2. Patients who have a confirmed or suspected hip fracture should have the following “Big Six” bundle of interventions carried out before leaving the ED (analgesia, early warning score, pressure area assessment, fluid assessment, bloods taken, cognitive assessment) 3. Every patient with hip fracture receives an “inpatient care bundle” within 24 h of admission (pressure area assessment, falls risk assessment, nutrition screen, full cognitive assessment) 4. Patients should undergo surgical repair of their hip fracture within 36 h of admission 5. No patient should be fasted for surgery repeatedly, and patients should be offered clear fluids orally up to 2 h before surgery 6. If patients are given a hemiarthroplasty, cemented implants are standard unless clinically indicated otherwise 7. Every patient identified as frail has a comprehensive geriatric assessment (CGA) performed within 3 days of admission 8. Patients are mobilized by the end of the first postoperative day (post-op day 1) and have a physiotherapy (PT) assessment by the end of the second postoperative day 9. Every patient is assessed by occupational therapy (OT) by the end of the third postoperative day 10. Every patient with hip fracture has bone health assessment or referral prior to leaving the orthopaedic ward 11. All patients have their recovery optimized such that they are discharged back to their original place of residence by day 30 after admission	

Adherence to each individual included standard was assessed on an all-or-none basis. However, for this study, standard 5 was split into separate standards for repeated fasting and for preoperative oral fluid intake. The cutoff for provision of oral fluids was set at ≤ 4 h prior to theatre because this was felt to represent a more realistic window in which to achieve this standard. Standard 8 was also divided into separate assessments for mobilization and physiotherapy respectively, since initial postoperative mobilization was often carried out by nursing staff. Standard 6 was excluded from the analysis because the use of hemiarthroplasty implants is appropriate for less than 50% of hip fracture patients. Standards 10 and 11 were not included due to their recent addition to the SSCHFP, with the available data too limited to provide reliable assessment.

### Demographic and patient variables

Data were gathered prospectively using a variety of sources such as patient medical notes and online validated reporting systems by trained LACs. Relevant demographics were collected including age, gender, pre-fracture residence, day of admission, time of admission, day of operation, and type of operation. Patients were categorized by day of admission according to each day of the week (Sunday to Monday between 00:00 and 23:59 h).

### Outcome variables

The primary outcome measure was the likelihood of adherence to the SSCHFP. Additional process measures of ‘weekend surgery’ and ‘operation type’ were also assessed. Patient outcomes were collected from electronic sources by LACs at 60 days post-admission, including post-discharge mortality.

### Statistical analysis

#### Day of admission

Descriptive analyses were performed to quantify the daily adherence to care standards for admission on each day of the week. The impact of the day of the week of admission on the included individual care standards was assessed using a multinomial logistic regression with adjustment for age, sex, and pre-fracture residence. Odds ratios (OR) with 95% confidence intervals (95% CI) and *p* values were produced, comparing each day of the week to an a priori determined reference day (Monday). Monday was chosen as the reference day due to its position as the beginning of the working week within Scotland. It was therefore presumed to enable good performance with respect to attainment of the SSCHFP, with no expected significant negative variance when compared with other weekdays.

**Fig. 1:**
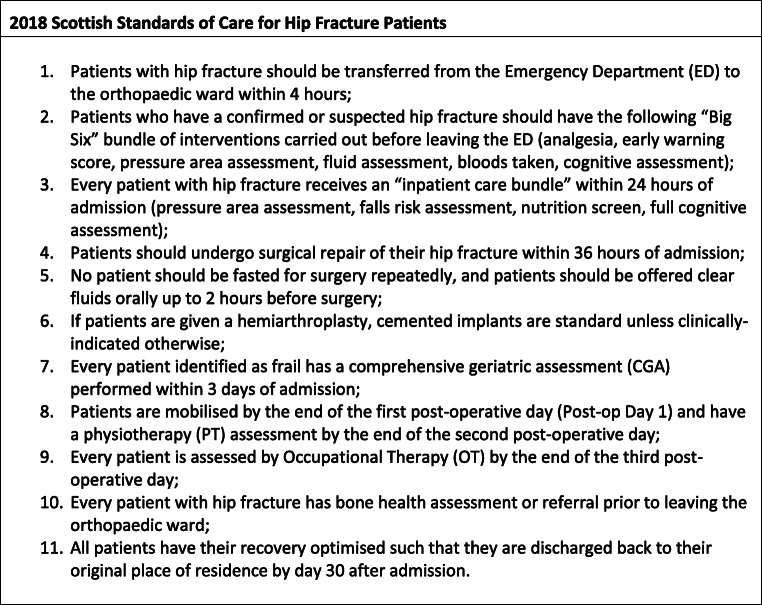
Comparative stratified cumulative attainment of SSCHFP according to day of the week

The impact of day of admission on cumulative standard adherence was also assessed. A cumulative score was produced from all of the included individual care standards giving a maximum value of 10 (full adherence) to a minimum of 0 (no adherence). Calculation of the median cumulative score was then performed, and results dichotomized to either higher or lower than the median value. This was utilized in a multinomial logistic regression to assess each day of admission with this binary cumulative score as a predictor, adjusted for age, sex, and pre-fracture residence, with comparison to the reference value. A total stratified score for each day of the week of admission was also produced (Fig. 1). This was performed by scoring each day of the week from 1 to 7 (7 = best; 1 = worst) for each individual care standard according to the OR. The scores for each day were then added across the 10 standards to give a total stratified score for each day.

All missing data were assumed missing at random as provisional checks did not reveal any significant confounding. All data analyses were performed using SPSS for Windows (version 24.0; IBM). The significance level for all reported analyses was set as *p* < 0.05.

#### Additional analyses

A post hoc analysis was undertaken to examine which days of the week patients underwent surgery and also met the standards of timely PT input, OT input, and CGA, respectively (if completed within the audit limit for that standard, which was within 4 days of surgery for PT/OT and 7 days post-admission for CGA). We compared the percentages (with 95% confidence intervals [95% CI]) for the day of the week upon which each standard was achieved (i.e. actual delivery of a Standard may occur on a different day from admission, but be classed as achieved if it occurred within the recommended time frame, e.g. occupational therapy assessment within 72 h of surgery).

### Ethics

Approval for the study was provided by NHS National Services, Scotland following review of the research protocol by the National Scottish Hip Fracture Committee. Data access was performed in compliance with the Caldicott Principles governing data utilization within the UK.

## Results

Of 15,351 eligible patients with hip fracture, 60-day post-admission data were available for 93.5% (*n* = 14,353), with 76.1% (*n* = 11,685) of individuals having information available for all of the included SSCHFP to allow for calculation of their cumulative care variable score. Of the total patients, 71.6% were female; 80–84 years age group was the largest age group (21.8%).

### Day of admission

#### Patient characteristics and outcomes

Upon which they were admitted, 15,351 patients (100%) had data pertaining to the day of the week. Patient characteristics for those admitted on each day of the week are presented in Table 2. There were no significant differences in patient age, sex, pre-fracture residence, and operation type for admission on any given day of the week.

**Table 2:** Comparative patient characteristics for those admitted on each day of the week with Chi^2^ analysis

Variable	Monday (*n* = %)	Tuesday (*n* = %)	Wednesday (*n* = %)	Thursday (*n* = %)	Friday (*n* = %)	Saturday (*n* = %)	Sunday (*n* = %)	*p* value
Age								
< 75	535 (24.6)	555 (24.2)	534 (24.2)	528 (23.3)	534 (24.3)	519 (24.6)	568 (27.0)	0.86
75–84	759 (34.9)	820 (35.8)	831 (37.7)	780 (34.4)	765 (34.7)	763 (36.2)	734 (34.9)	
≥ 85	880 (40.5)	914 (39.9)	841 (38.1)	957 (42.3)	903 (41.0)	828 (39.2)	803 (38.1)	
Sex								
Male	639 (29.4)	702 (30.7)	613 (27.8)	608 (26.8)	634 (28.8)	577 (27.3)	594 (28.2)	0.08
Female	1535 (70.6)	1587 (69.3)	1593 (72.2)	1657 (73.2)	1568 (71.2)	1533 (72.7)	1511 (71.8)	
Pre-fracture residence								
Home/sheltered	1584 (72.9)	1715 (75.0)	1652 (74.9)	1617 (71.4)	1615 (73.3)	1563 (74.1)	1562 (74.2)	0.09
Other	590 (27.1)	590 (25.0)	554 (25.1)	648 (28.6)	587 (26.7)	547 (25.9)	542 (25.8)	
Weekend operation?								
Yes	16 (0.8)	50 (2.2)	139 (6.4)	514 (23.3)	1573 (72.8)	1379 (66.6)	328 (15.9)	< 0.001
No	2093 (99.2)	2173 (97.8)	2019 (93.6)	1696 (76.7)	588 (27.2)	693 (33.4)	1736 (84.1)	
Operation type								
Surgical fixation	484 (45.6)	561 (48.0)	489 (44.3)	508 (45.4)	531 (47.5)	470 (45.5)	491 (47.8)	0.29
Hemiarthroplasty	522 (49.2)	539 (46.1)	539 (48.8)	542 (48.5)	530 (47.4)	511 (49.4)	484 (47.1)	
Total hip replacement	55 (5.2)	68 (5.8)	76 (6.9)	68 (6.1)	56 (5.0)	53 (5.1)	52 (5.1)	

#### The influence of day of admission on individual standard attainment

**Fig. 2:**
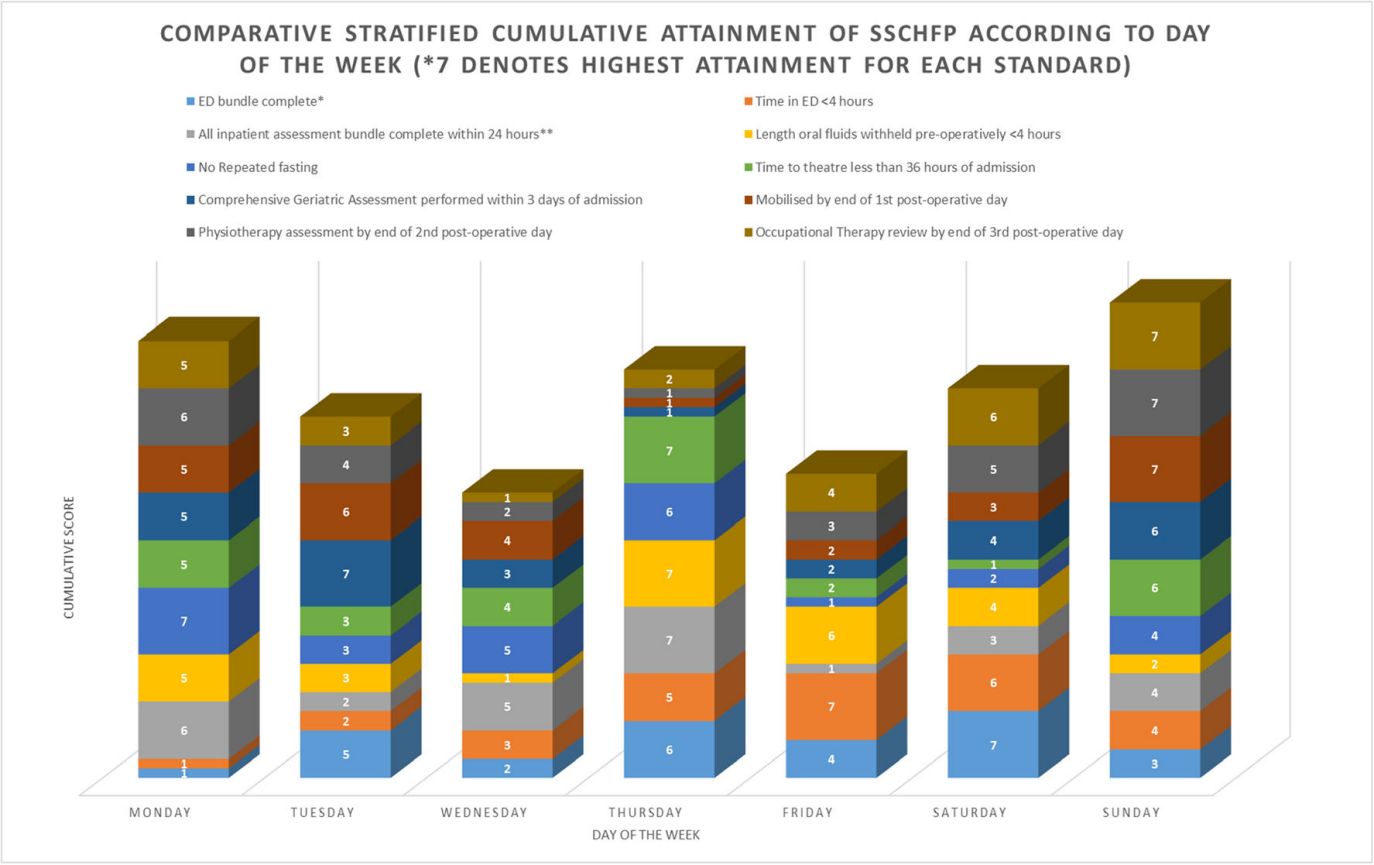
Graph to show % achievement of each of the standards of care by day of admission

We used a multinomial regression model to assess the influence of the day of admission on individual care standard attainment, with adjustment for age, sex, and pre-fracture residence. These are detailed in Table 3. A visual representation of individual standard attainment by day of admission is shown in Fig. 2.

**Table 3:** Multinomial logistic regression with Odds ratios and confidence intervals regarding individual care standards for those admitted during each day of the week compared with reference value (Monday) including adjustment for age, sex, and location of residence prior to admission

Variable	Odds ratio (95% CI), *p* value					
	Tuesday	Wednesday	Thursday	Friday	Saturday	Sunday
Not all of ED Big 6 complete*	0.96 (0.82 to 1.13), *p* = 0.63	0.97 (0.83 to 1.15), *p* = 0.76	0.95 (0.81 to 1.11), *p* = 0.53	0.97 (0.82 to 1.14), *p* = 0.69	0.92 (0.79 to 1.09), *p* = 0.35	0.97 (0.82 to 1.15), *p* = 0.73
Time in ED > 4 h	0.97 (0.79 to 1.19), *p* = 0.76	0.94 (0.77to 1.16), *p* = 0.56	0.83 (0.67 to 1.03), *p* = 0.09	0.73 (0.59 to 0.91), *p* = 0.01	0.78 (0.63 to 0.97), *p* = 0.02	0.85 (0.69 to 1.10), *p* = 0.14
All inpatient assessment bundle not complete within 24 h**	1.06 (0.93 to 1.21), *p* = 0.37	1.01 (0.89 to 1.16), *p* = 0.87	0.99 (0.86 to 1.13), *p* = 0.84	1.07 (0.93 to 1.22), *p* = 0.34	1.06 (0.93 to 1.21), *p* = 0.39	1.03 (0.90 to 1.18), *p* = 0.62
Time oral fluids withheld preoperatively > 4 h	1.00 (0.88 to 1.14), *p* = 0.95	1.06 (0.92 to 1.21), p = 0.44	0.97 (0.85 to 1.11), *p* = 0.97	0.99 (0.86 to 1.13), *p* = 0.82	1.00 (0.88 to 1.15), *p* = 0.96	1.01 (0.89 to 1.16), *p* = 0.85
Repeated fasting	1.18 (1.02 to 1.37), *p* = 0.03	1.07 (0.92 to 1.25), p = 0.37	1.06 (0.91 to 1.24), p = 0.44	1.22 (1.05 to 1.42), *p* = 0.009	1.22 (1.05 to 1.41), *p* = 0.01	1.17 (0.96 to 1.30), *p* = 0.16
Time to theatre more than 36 h of admission	1.12 (0.99 to 1.28), *p* = 0.08	1.05 (0.92 to 1.20), *p* = 0.44	0.95 (0.83 to 1.09), *p* = 0.46	1.24 (1.09 to 1.41), *p* = 0.001	1.23 (1.08 to 1.41), *p* = 0.002	0.95 (0.83 to 1.09), *p* = 0.44
Comprehensive geriatric assessment not performed within 3 days of admission	0.91 (0.79 to 1.04), *p* = 0.15	1.17 (1.03 to 1.33), *p* = 0.02	1.88 (1.65 to 2.13), *p* < 0.001	1.41 (1.24 to 1.61), *p* < 0.001	1.01 (0.88 to 1.16), *p* = 0.91	0.94 (0.82 to 1.08), *p* = 0.36
Not mobilized by end of first postoperative day	0.98 (0.86 to 1.12), *p* = 0.78	1.09 (0.95 to 1.24), *p* = 0.21	1.77 (1.56 to 2.01), *p* < 0.001	1.48 (1.30 to 1.69), *p* < 0.001	1.13 (0.99 to 1.29), *p* = 0.07	0.87 (0.76 to 0.99) *p* = 0.04
Physiotherapy assessment not performed by end of second postoperative day	1.86 (1.41 to 2.45), *p* < 0.001	4.24 (3.29 to 5.47), *p* < 0.001	8.61 (6.75 to 10.99), *p* < 0.001	3.47 (2.68 to 4.49), *p* < 0.001	1.41 (1.05 to 1.89), *p* = 0.02	1.02 (0.74 to 1.40), *p* = 0.90
Occupational therapy review not performed by end of third postoperative day	1.11 (0.97 to 1.26), *p* = 0.13	1.40 (1.23 to 1.59), *p* < 0.001	1.29 (1.31 to 1.47), *p* < 0.001	1.01 (0.89 to 1.16), *p* = 0.83	0.89 (0.78 to 1.02), *p* = 0.09	0.86 (0.75 to 0.98), *p* = 0.02

OR = Odds ratio, 95% CI = 95% Confidence interval. *Big six ED bundle includes analgesia given, blood tests performed, optimization of fluid balance, pressure area assessment, vital signs recorded, and delirium screening. **Inpatient assessment bundle includes formal cognitive assessment; fluid, food and nutrition assessment; pressure area assessment (Waterlow scoring); falls risk assessment; and MDT care

We found a significantly lower probability of time spent in ED to be more than 4 h for Friday and Saturday admissions compared with the reference value (Monday). Patients admitted on a Tuesday, Friday, and Saturday were significantly more likely to undergo repeated fasting. Those admitted on Friday and Saturday were also more likely than those admitted on Monday to wait more than 36 h for surgery. Individuals admitted on a Thursday were nearly twice as likely as the reference value to not receive a CGA within 3 days of admission and were less likely to undergo OT assessment by the end of the third postoperative day (POD). Thursday admissions were also more likely not to be mobilized by the end of the first postoperative day and more than eight times more likely to not have a physiotherapy assessment performed by the end of the second postoperative day compared with Monday admission. Compared with the reference, Friday admission was associated with a higher probability of not having a CGA within 3 days of admission, higher chance of not being mobilized by the end of the first postoperative day, and a greater probability of not being assessed by physiotherapy by the end of postoperative day 2. Individuals admitted on a Sunday were more likely to be mobilized by the end of the first postoperative day and undergo OT assessment by the third postoperative day in comparison to Monday.

#### The influence of day of admission on cumulative standard attainment

Results for the influence of day of admission on cumulative standard attainment are shown in Table 4. Admission on a Thursday was associated with the highest chance of obtaining a below median score for cumulative standard attainment compared with the reference value (Monday). Admission on Wednesday, Friday, and Saturday was also associated with a significantly greater likelihood of not obtaining the median value for cumulative standard attainment.

**Table 4:** Multinomial logistic regression with odds ratios and confidence intervals regarding attainment of below median cumulative care standard score for those admitted during each day of the week compared with reference value (Monday) including adjustment for age, sex, and location of residence prior to admission

Day of admission	Cumulative standard attainment below median value (OR)	95% CI	*p* value
Tuesday	1.11	0.97 to 1.27	0.14
Wednesday	1.35	1.18 to 1.55	**<** 0.001
Thursday	1.88	1.63 to 2.16	**<** 0.001
Friday	1.64	1.43 to 1.89	**<** 0.001
Saturday	1.31	1.14 to 1.50	**<** 0.001
Sunday	0.98	0.85 to 1.12	0.74

In addition, we performed assessment for stratified cumulative attainment of the SSCHFP according to day of the week of admission. Sunday was associated with the highest score, with Wednesday the lowest stratified attainment score (Fig. 1).

### Additional analyses

We assessed the level of achievement of standards on each day of the week (rather than assessing standard achievement for patients admitted on each day). There was a larger number of inpatients who met the following standards on a weekday (Monday to Friday) than a weekend (Saturday and Sunday): Physiotherapy by POD2 (pooled mean per day 16.8% [95% CI 16.2% to 17.2%] versus 7.90% [95% CI 7.45 to 8.33%], respectively); OT assessment by POD3 (pooled mean per day 18.1% [95% CI 17.3% to 18.9%] versus 4.68% [95% CI 4.27% to 5.09%], respectively); and CGA within 3 days of admission (pooled mean per day 19.3% [95% CI 18.5% to 20.1%] versus 1.78% [95% CI 1.53% to 1.93%], respectively). There was also a difference in the number of patients undergoing an operation from Monday to Friday compared with Saturday and Sunday (pooled mean per day 14.7% [95% CI 14.1% to 15.3%] versus 13.3% [95% CI 12.8% to 13.8%], respectively).

## Discussion

To the best of our knowledge, this is the first study to examine how the day of admission to hospital influences overall quality of patient care in hip fracture. Previous studies have examined the association between hip fracture outcomes, such as mortality and length of stay, and the so called ‘weekend effect’ with varied results [10–12, 19]. We found significant differences in the quality of care provided to hip fracture patients depending on the day of admission, as evidenced by statistically significant discrepancies in adherence to the nationally agreed quality standards (SSCHFP). Our analysis provides evidence to suggest that reorganization of services, particularly relating to Allied Health Professional and specialist services for older people, may be required in order that all patients receive equitable care irrespective of the day they are admitted to a hospital.

Regarding quality of care and standard attainment, Thursday and Friday admission was associated with poorer quality of care. This included a lower probability of meeting the median cumulative standard attainment and a significantly reduced chance of receiving timely geriatric care, prompt postoperative mobilization, PT, and OT input. It is likely that this is due to a delayed process of care for these patients (who typically undergo surgery on a Friday) as a consequence of limited Allied Health Professional (AHP) and geriatrician availability Saturday and Sunday. This is supported by our finding that care standards relating to AHP and geriatric input were significantly less likely to be met on a Saturday and Sunday compared with Monday to Friday.

Improving Saturday and Sunday AHP services may facilitate an improvement in quality of care in line with the national care guidelines, as well as yield better patient outcomes [8]. Previous research has shown that the availability of weekend physiotherapy services is variable [20], but that these services have the potential to reduce length of stay and improve functional recovery in hip fracture patients [21]. There is also an association between the intensity and frequency of geriatrician input and improved survival of patients with hip fracture [22–24]. The comparatively greater adherence to the care standards that we observed on Sundays and at the start of the working week may reflect the greater availability of resources at these times and an increased focus on providing these services early in the week (e.g. Monday) to accommodate patients who were admitted over the weekend.

The increased time to theatre and likelihood of repeated fasting observed for patients admitted on a Friday or Saturday is likely related to the reduced availability of preoperative standard resources on Saturday and Sunday and the lack of dedicated trauma operating lists on these days in many hospital serving as a barrier to the prompt surgical treatment of hip fractures [25]. This was consistent with the study findings regarding a lower probability of patients attending theatre on Saturday and Sunday in our additional analysis. There is evidence demonstrating that the introduction of Saturday and Sunday orthopaedic trauma theatre sessions significantly reduces time to surgery and reduces length of hospital admission [26].

Adherence to the ED Big 6 standard (achieving all 6 constituent parts) was noted to be poor across the working week. Further investigation delineating each of the components of the ED Big 6 should be undertaken to determine if it is one particular aspect of this standard that is problematic, and how this varies between hospitals, in an attempt to improve future adherence.

There are some limitations to the study, the main point of which was the inability to include confounders not currently collected by the LACs that may influence standard adherence, such as hospital-wide resource and flow information, in-hospital complications, or other case-mix factors. This includes potential variations in service delivery according to the hospital size and status (e.g. Major Trauma Centre), location, and catchment indices of deprivation. Further research including these factors is warranted given the potential influence of these larger service provision factors on healthcare delivery and outcomes. The data collection by LACs was subject to these posts being filled, and thus, there may be missing data from these periods. Nonetheless, the data collected by LACs represent a large majority of Scottish hip fracture patients admitted to surgical hospitals (e.g. 90% of those from May 2016 to December 2017 when collection was full-time). Rigorous data validation was performed on a regular basis to ensure that any potential irregularities were minimized, and all data were collected according to strict guidelines. The SSCHFP have been revised during the course of the data collection period, and we used the SSCHFP in their current form to provide the most up-to-date reflection of best quality care. Data were incomplete for some standards (due to recent inclusion as part of the SSCHFP) and significant ineligibility of a large proportion of patients to other standards (e.g. Standard 6, where only 48% patients included underwent hemiarthroplasty). Whilst this slightly restricts the conclusions that can be drawn compared with the full set of SSCHFP, it was felt that this was significantly better than removing large quantities of valuable data which would have negatively influenced the power of the study.

Though this study did not examine the association between quality of care measures and patient outcomes within this setting, our previous work has displayed a connection between low adherence to care standards and patient health [8]. There is therefore indirect evidence for a relationship between day of admission-related discrepancies in quality of care and outcomes such as mortality, length of stay, and discharge destination. Further work to directly correlate admission-related variation and patient outcomes would be beneficial in providing impetus for change in weekend service provision, in addition to that already provided from an ethical and humanitarian perspective.

## Conclusion

We found evidence that hip fracture patients admitted Thursday or Friday receive a lower quality of care with respect to the Scottish Standards of Care for Hip Fracture Patients. Our findings suggest that this relates to limited provision of services on Saturday and Sunday that are required for post-surgical standard attainment, particularly physiotherapists, occupational therapists, and orthogeriatricians. For the first time in the literature, we demonstrate that the specific day of admission, rather than groupings such as ‘weekend’ or ‘weekday’, should be utilized to provide better understanding of the relationship between timing of admission, processes of care, and quality outcomes. The incidence of hip fracture is projected to increase dramatically alongside the ageing population. Reducing variation in the standard of care provided is key to providing high-quality, safe, equitable care to all patients. There is an ongoing clinical and health economic need to assess the efficiency and efficacy of care provided to hip fracture patients in order to inform service organization and appropriate allocation of resources. Our findings are of significant import to healthcare organizations, personnel, and administrators responsible for the provision of hip fracture services globally.
